# TNF-Related Apoptosis Inducing Ligand in Ocular Cancers and Ocular Diabetic Complications

**DOI:** 10.1155/2015/424019

**Published:** 2015-03-05

**Authors:** Paolo Perri, Giorgio Zauli, Arianna Gonelli, Daniela Milani, Claudio Celeghini, Giuseppe Lamberti, Paola Secchiero

**Affiliations:** ^1^Department of Biomedical and Specialist Surgical Sciences, University of Ferrara, 44121 Ferrara, Italy; ^2^Institute for Maternal and Child Health-IRCCS “Burlo Garofolo”, 34137 Trieste, Italy; ^3^Department of Morphology, Surgery and Experimental Medicine and LTTA Centre, University of Ferrara, 44121 Ferrara, Italy; ^4^Department of Life Sciences, University of Trieste, 34137 Trieste, Italy

## Abstract

TNF-related apoptosis inducing ligand (TRAIL) is an intensively studied cytokine, in particular for its anticancer activity. The discovery that conjunctival sac fluid contains extremely high levels of soluble TRAIL as compared to other body fluids suggested important implications in the context of the immunological surveillance of the eye, in particular of the anterior surface. In this review, we discuss the potential physiopathologic and therapeutic role of the TRAIL/TRAIL receptor system in a variety of ocular cancers. Moreover, since an increasing amount of data has indicated the important biological activities of the TRAIL/TRAIL receptor systems also in a completely different pathologic context such as diabetes mellitus, in the second part of this review we summarize the currently available data on the involvement of TRAIL in the ocular complications of diabetes mellitus as modulator of the inflammatory and angiogenic response in the eye.

## 1. The TRAIL/TRAIL Receptor System

TNF-related apoptosis inducing ligand (TRAIL), also known as Apo2 ligand or TNFSF10, was originally discovered independently by Ashkenazi's group at Genentech [1] and Goodwin's group at Immunex [2] almost 20 years ago. Subsequently, the complex system of transmembrane TRAIL receptors was identified and it resulted to be composed of TRAIL-R1/death receptor 4, TRAIL-R2/death receptor 5, TRAIL-R3/decoy receptor 1, and TRAIL-R4/decoy receptor 2 [3]. Similarly to other members of the TNF superfamily, TRAIL acts as homotrimeric molecule, but it has the unique property of containing a central zinc atom at the trimer interface, which is important for the solubility, stability, and biological activity of TRAIL.

The first and best characterized activity of TRAIL is to promote apoptosis in a variety of cancer cell types by interacting with the so-called death receptors TRAIL-R1 and TRAIL-R2 [3]. On the other hand, the two additional transmembrane receptors TRAIL-R3 and TRAIL-R4 as well as the soluble receptor osteoprotegerin (OPG) have been proposed to act as decoy or regulatory receptors [4, 5]. The biological activity of TRAIL on cancer cells is mediated by the activation of the extrinsic pathway of apoptosis induced by clustering (homotrimerization) of TRAIL-R1 or TRAIL-R2 at the cell surface level. This event promotes receptor binding to the adaptor protein Fas-associated death domain (FADD), which in turn recruits the initiator caspases 8 and 10 and leads to the assembling of the death-inducing signaling complex (DISC). DISC formation is modulated by several inhibitory mechanisms, such as (i) at intracellular level by the caspases 8/10 inhibitor c-FLIP, which directly interferes with receptor activation; (ii) at membrane level, due to the role of the decoy receptors TRAIL-R3 and TRAIL-R4, which can heterotrimerize with TRAIL-R1 and TRAIL-R2 and attenuate, or even neutralize, the proapoptotic signal. Additional mechanisms of regulation of TRAIL-mediated apoptosis have been discovered and consist in posttranslational modifications of TRAIL-R1 and TRAIL-R2, subsequent to glycosylation and palmitoylation [6]. Also the lectin galectin-3, which recognizes multiple N-acetyllactosamine sequences, when highly expressed shows the property to immobilize death receptors by trapping them in a nanocluster lattice, blocking DISC formation [7].

In spite of its initial characterization in the cancer biology field, our group have provided robust experimental evidence that TRAIL can drive prosurvival and/or proliferative effects and can modulate differentiation in normal cell types such as human intestinal cells [8], bone marrow multipotent stromal cells [9], myeloid cells [10–12], and osteoclastic cells [13, 14]. These trophic effects of TRAIL are mediated by the activation of the PI3K/Akt and ERK pathways [15, 16] and of the transcription factor NF-kB [17, 18] in a cell type specific context. Therefore, the biologic outcome of TRAIL stimulation seems to be determined in each cell type by the balance of different elements, comprising transmembrane receptor expression (including density and localization), and intracellular components that, eventually, could switch the cell phenotype either from apoptotic-prone to apoptotic-resistant or vice versa [19]. For instance, sensitization to TRAIL-induced apoptosis can also be derived from the activation of the p53 pathway. Indeed, several studies have shown that activation of the p53 pathway elicited by the nongenotoxic small molecule inhibitor of MDM2 Nutlin-3 upregulates the expression of TRAIL-R2 [20, 21], which in turn can be sensitized to TRAIL-mediated apoptosis [22]. Interestingly, however, the combination of Nutlin-3 and recombinant TRAIL is much less toxic in normal cells with respect to cancer cells, further underlining the complexity of the regulation of TRAIL biological effects at the intracellular level.

## 2. TRAIL Expression in Cornea and in Conjunctival Sac

Significant levels of TRAIL transcripts and proteins have been detected in many human tissues [23] including normal epithelia such as skin [24, 25], buccal mucosa [26], intestinal mucosa [8], and corneal and conjunctival epithelia [27–29]. In particular, expression of TRAIL has been previously documented in human corneal and conjunctival epithelia by* in situ* immunohistochemistry using a monoclonal antibody which is highly specific for TRAIL [28]. While corneal epithelium was strongly positive for TRAIL expression with a more intense TRAIL immunostaining observed in the basal layers, conjunctival epithelium expressed TRAIL but at a lower intensity with respect to the corneal epithelium [28]. On the other hand, no TRAIL immunostaining was documented in sclera [28]. The immunohistochemistry data were confirmed by flow cytometry in primary corneal epithelial cells and were obtained from patients undergoing photorefractive keratectomy [28]. Such a widespread distribution of TRAIL expression differs from that of most TNF family members and is in line with the* in vitro* data, showing that TRAIL is not cytotoxic on most normal tissues* in vivo*. Of note, in the same study [28], we have also demonstrated that the conjunctival sac fluid contained the highest amount of soluble TRAIL ever measured in a body fluid. Indeed, the mean level of soluble TRAIL detected in the conjunctival sac fluid was 26 ng/mL, >200-fold greater than that found in human serum/plasma (range of 55 to 110 pg/mL). It is particularly remarkable that the concentrations of TRAIL in the conjunctival sac fluid were in range able to significantly induce apoptosis* in vitro* in B lymphoma cells [28].

## 3. TRAIL and Ocular Tumors

In spite of the lack of immune cells, the eye is refractory to the development of tumors (both primary intraocular tumors and tumors metastasizing from distant sites). In particular, corneal and conjunctival tumors are extremely rare [30]. Thus, a reasonable hypothesis based on the extremely high levels of TRAIL in the conjunctival sac fluid [28] is that TRAIL is involved in the antitumoral surveillance of the anterior epithelium of the ocular globe. In this respect, it is interesting to analyze the available data concerning the potential susceptibility of ocular tumors to TRAIL-based therapy.

### 3.1. Rhabdomyosarcoma

**Figure 1:**
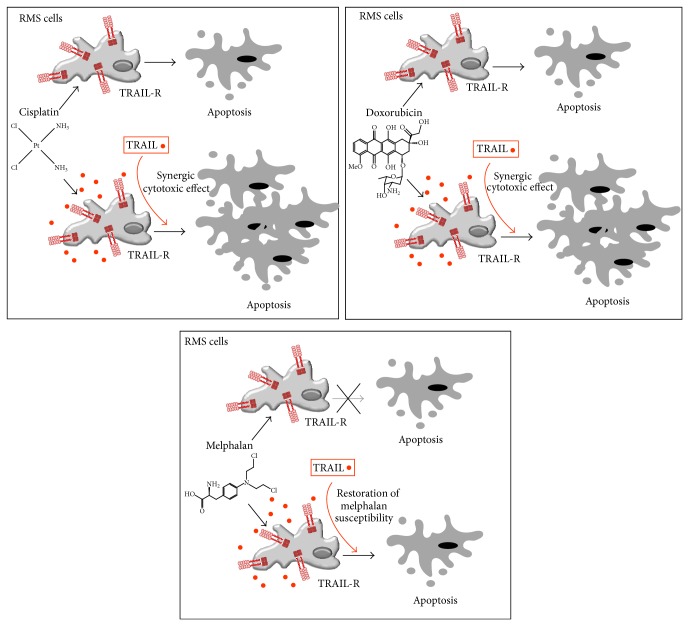
Synergic therapeutic approaches between TRAIL and chemotherapeutic drugs on rhabdomyosarcoma (RMS). Antitumor therapies based on the combination of standard chemotherapy with TRAIL treatment can lead to improved RMS cell apoptosis and/or to the overcoming of chemotherapy resistance. TRAIL-R, TRAIL receptors.

Rhabdomyosarcoma (RMS) represents the most common soft tissue sarcoma in children and nearly 35–40% of RMS occurs in the head and in the neck including orbital tissue. This highly malignant tumor is insensitive to chemotherapy alone and, thus, TRAIL represents a promising candidate for new options in combined therapy. Indeed, when used together with cisplatin, TRAIL has shown synergistic cytotoxic effects on RMS cells [31], which was related to the downregulation of mitochondrial membrane potential in RMS cells. TRAIL also showed additive cytotoxic effects on RMS cells when used in combination with doxorubicin [32]. Finally, TRAIL was able to restore the susceptibility of melphalan-resistant RMS tumor cells to cytotoxicity mediated by caspases 2 and 3 [33]. Overall, these data clearly indicate that TRAIL is potentially active in RMS in combination with different chemotherapeutic drugs which are currently employed in the treatment of RMS (Figure 1).

### 3.2. Uveal Melanoma

**Figure 2:**
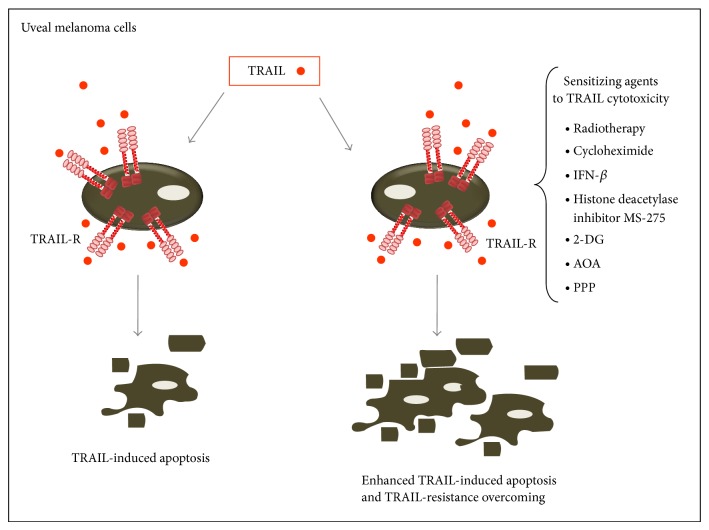
Potential TRAIL-mediated therapeutic approaches to target uveal melanoma cells. Different agents such as radiotherapy, cycloheximide, interferon-beta (IFN-*β*), class-I specific histone deacetylase inhibitor MS-275, 2-deoxy-D-glucose (2-DG), the glutamate-dependent transaminase inhibitor aminooxyacetate (AOA), and the specific IGF-1R kinase inhibitor cyclolignan picropodophyllin (PPP) may sensitize uveal melanoma cells to TRAIL-mediated apoptosis or enhance TRAIL cytotoxicity. TRAIL-R, TRAIL receptors.

Uveal melanoma is a common ophthalmic malignancy in human which is prone to metastatize to other organ such as liver [34]. The prognosis of patients carrying uveal melanoma is quite poor and the mean overall survival is generally 2–8 months, and the survival of patients that suffer from metastasis benefits little from the current treatments, including photocoagulation, surgery, and radiotherapy. Therefore, alternative therapeutic options are urgently needed to treat malignant uveal melanoma. Among the different experimental approaches explored, several studies have investigated the potential therapeutic effects of TRAIL (Figure 2). In this respect, it has been proposed that radiotherapy might represent an important tool for sensitizing uveal melanoma to TRAIL cytotoxicity [35] and the study of Ren et al. [36] was the first one showing a potential efficacy of TRAIL in promoting apoptosis of uveal melanoma cells. Moreover, cycloheximide exerted a profound effect in enhancing TRAIL-induced apoptosis in uveal melanoma cell lines, including metastatic cell lines [36]. Similarly, interferon-*β* also produced an enhancement of TRAIL-induced apoptosis even in melanoma cell lines that were previously shown to be resistant [36]. These data might also account for the finding that, in preclinical studies, interferon- (INF-) *β* induced (i) the serum release of TRAIL and (ii) cell apoptosis and antitumor effects against melanoma [37]. More recently, an approach based on the adenovirus-based TRAIL expression has been proposed to treat malignant uveal melanoma with high cell specificity [38]. Interestingly, in a recent study, it has also been shown that while TRAIL-R1/DR4 and TRAIL-R2/DR5 methylation is not frequent in cutaneous melanoma, it was very frequent in uveal melanoma [39]. Consistently, the same group of investigators has demonstrated that class I-specific histone deacetylase inhibitor MS-275 overrides TRAIL resistance in melanoma cells by downregulating c-FLIP [40]. Moreover, different studies have shown that sensitivity to TRAIL-induced apoptosis of cultured melanoma cells may be restored/enhanced by exposure to 2-deoxy-D-glucose (2-DG) [41, 42], aminooxyacetate (AOA), an inhibitor of glutamate-dependent transaminase [43], or cyclolignan picropodophyllin (PPP), a specific inhibitor of IGF-1R kinase activity [44].

### 3.3. Orbital Lymphoma

Orbital lymphoma accounts for 10% of the orbital malignant tumors [45, 46]. Interestingly, it has been shown that the combination of irradiation and TRAIL synergistically induced apoptosis in malignant lymphatic cell lines enhances the efficacy of combined tumor therapy in the radiotherapy-tolerant lymphatic cells with Bcl-2 overexpression [46]. Of note, Unnithan and Macklis documented an increase of TRAIL expression in patients treated with radiation for lymphoma [47]. Moreover, a number of studies of our group and other groups of investigators have addressed the role of individual TRAIL receptor in mediating the proapoptotic activity of ionizing radiation in different types of malignancies, including lymphoblastic cells, documenting that TRAIL-R1/DR4 represents the principal receptor involved in mediating the proapoptotic activity of ionizing radiation [48–51].

## 4. TRAIL and Ocular Complications of Diabetes Mellitus

Beside the well-studied role of TRAIL in oncologic settings, an increasing amount of data has indicated the important biological activities of the TRAIL/TRAIL receptor systems also in diabetes mellitus [52–54]. For the purpose of the present review, we summarize the currently available data on the involvement of TRAIL in the ocular complications associated to diabetes mellitus [55].

### 4.1. Proliferative Diabetic Retinopathy

Proliferative diabetic retinopathy, as well as other proliferative retinopathies, is caused by widespread ischemia of the inner retinal layers, secondary to closure of the parts of the retinal capillary bed, leading to new vessel formation in the retina. Proliferative diabetic retinopathy is considered a wound healing-like response in which neovascularization is accompanied by an influx of inflammatory cells and the development of myofibroblasts, with fibrovascular contraction causing hemorrhages, retinal detachment, and blindness [55]. Vascular endothelial growth factor (VEGF) and other proangiogenic factors produced by the ischemic retina are considered to be the major causal growth factors in the neovascularization process. However, negative regulators of angiogenesis, such as pigment-epithelium derived factors (PEDF), might also interfere with the VEGF-angiogenesis by downregulating the PI3K/Akt pathway. In this context, an interesting study demonstrated that PEDF, which is an intrinsic antiangiogenic factor and a potential antitumor agent, increases the expression of TRAIL [56]. Moreover, a recent study suggested that TRAIL might play an important role in restraining endothelial cell proliferation in the retina, as: (i) TRAIL deficient mice exhibited delayed regression of retinal neovascularization and (ii) recombinant TRAIL-induced apoptosis of retinal endothelial cells [57]. In this respect studies by Secchiero's group [58, 59] and other investigators [60] have suggested that TRAIL plays an important role in modulating vascular function and might have prognostic value in diabetic patients affected by cardiovascular complications [61–63]. Therefore, the high levels of soluble TRAIL documented in the conjunctival sac fluid may suggest an important role of TRAIL also in modulating the inflammatory and angiogenic response in the eye [64]. In this respect, we have also documented that the levels of TRAIL in the conjunctival sac fluid are significantly decreased in patients affected by proliferative retinopathy [29]. Although the relationship between external soluble TRAIL (released in the conjunctival sac fluid) and internal TRAIL (expressed in the retina or vitreous body) is not known, these data strengthen the notion that TRAIL plays an important anti-inflammatory role and antiangiogenic role for the anatomic and functional stability and for the safety of the ocular surfaces. Thus, it is plausible to suppose that a decreased production and/or release of TRAIL might contribute to worsening proliferative diabetic retinopathy by reducing the degree of apoptosis in retinal endothelial cells. In line with a potential role of TRAIL in mediating antiangiogenic activity, it has been shown that TRAIL might play an important role also in the regulation of retinal neovascularization (RNV) [57]. In particular, while RNV naturally regressed in wild-type mice, TRAIL(−/−) mice continued to display significantly high levels of RNV attributed to a significant decrease in neovascular tuft cells undergoing apoptosis. Of interest, the antiangiogenic activity of TRAIL has been suggested also in recent studies in patients affected by colon cancer under treatment with bevacizumab [65, 66] and confirmed in* in vitro* studies [67, 68].

### 4.2. Age-Related Macular Degeneration

An important role of apoptosis driven by TRAIL also in age-related macular degeneration (AMD), an important ocular disorder which represents a leading cause of irreversible vision loss in elderly, with diabetes playing a role as risk factor for AMD, has been proposed. Indeed, in a large cohort study consisting of 1826 subjects which included 300 cases with AMD, a significant association between TRAIL-R1 TNFRSF10-LOC389641 rs13278062 and increased risk of late macular age-related degeneration was observed [69]. Moreover, Anand et al. [70] have found significantly lower levels of TRAIL-R3 in serum samples of patients affected by AMD compared to controls. Since TRAIL-R3 is believed to act as dominant-negative receptor, the authors suggest that the low levels of TRAIL-R3 in these patients may increase the amount of TRAIL interacting with the proapoptotic receptors (TRAIL-R1 and TRAIL-R2), thus resulting in enhanced TRAIL apoptosis of photoreceptors and retinal pigment epithelium cells, which are well known phenomena involved in the pathogenesis of AMD [71].

## 5. Conclusions

The TRAIL/TRAIL-R system plays an important role in relevant ocular pathologic settings, such as cancers and major diabetic complications (id, retinopathy, and macular degeneration). A central finding was the discovery that the expression/release of TRAIL in different ocular tissues is extremely elevated. Thus, TRAIL likely represents a major actor involved in the immune surveillance against ocular tumours as well as modulator of the inflammatory and angiogenic response in the eye. Indeed, besides its well known ability to induce apoptosis in tumor cells, a role of TRAIL/TRAIL receptors in endothelial cell biology has recently emerged, revealing a trophic effect of TRAIL on primary normal endothelial cells and antiangiogenic activity in a variety of pathologic experimental conditions. The antiangiogenic function of TRAIL might represent a common link between the apparently distinct roles of TRAIL in cancer and in endothelial cell biology.

## Abbreviations

2-DG: 2-Deoxy-D-glucose
AMD: Age-related macular degeneration
DISC: Death-inducing signaling complex
ERK: Extracellular signal-regulated kinases
FADD: Fas-associated death domain
INF: Interferon
MDM2: Mouse double minute 2 homolog
NF-kB: Nuclear factor kappa-light-chain-enhancer of activated B cells
OPG: Osteoprotegerin
PEDF: Pigment epithelium-derived factor
RMS: Rhabdomyosarcoma
RNV: Retinal neovascularization
TRAIL: TNF-related apoptosis inducing ligand
VEGF: Vascular endothelial growth factor.
